# Habitat-based amide proton transfer-weighted MRI model for predicting BRAF mutation and prognostic stratification in rectal cancer

**DOI:** 10.3389/fonc.2026.1862322

**Published:** 2026-07-03

**Authors:** Li Zhang, Longchao Li, Zhaojun Ren, Yaxin Niu, Jing Zhang, Xiaoyan Lei

**Affiliations:** Department of MRI, Shaanxi Provincial People’s Hospital, Xi’an, China

**Keywords:** amide proton transfer imaging, BRAF, diffusion-weighted imaging, prognosis, rectal cancer

## Abstract

**Objectives:**

This study aims to investigate the efficacy of a novel habitat-based histogram features derived from amide proton transfer-weighted (APTw) MRI for predicting BRAF mutation status in RC, compared and combined with diffusion-weighted imaging (DWI), and the potential for prognostic stratification.

**Methods:**

This study prospectively enrolled 269 patients with RC from June 2021 to August 2025, divided into a training set (n=188) and a testing set (n= 81) using a fixed random seed. According to BRAF status, patients were categorized into a wild-type group (n=214) and a mutant group (n=55). K-means clustering was applied to partition the tumors into 7 sub-regions, from which corresponding histogram features of APTw and apparent diffusion coefficient (ADC) maps were extracted. The extracted features were subsequently filtered using stepwise regression. The predictive performance of five models—namely, the habitat-based APTw model, habitat-based ADC model, habitat-based APTw+ADC combined model, clinical factors model, and the comprehensive model integrating all features—was evaluated using receiver operating characteristic (ROC) curve analysis and decision curve analysis (DCA). Additionally, Kaplan-Meier survival curves, constructed based on the combined nomogram, were used to assess 2-year disease-free survival (DFS) in the entire cohort.

**Results:**

The K-means model with K = 7 demonstrated optimal predictive power for BRAF mutation, outperforming models with K = 5 or 6, with the mutant group showing significant differences in APT/ADC histogram features in specific sub-regions compared to the wild-type group. The nomogram integrating APTw/ADC subregion histogram features and the clinical factors showed superior discrimination, achieving an AUC of 0.83 (95% CI: 0.707-0.952), which was significantly higher than that of the habitat-based APTw model (0.733), habitat-based ADC model (0.71), or clinical factors model (0.661) (all *P* < 0.05) in the testing set. Furthermore, patients stratified into the mutant group by the nomogram had a significantly worse 2-year disease-free survival (DFS) than those in the wild-type group (*P* < 0.05).

**Conclusion:**

Habitat-based APT/ADC histogram features combined with clinical model showed good performance in predicting BRAF mutation and DFS of RC patients, which could provide valuable information for individualized treatment.

## Introduction

Rectal cancer (RC) ranks among the most common malignant tumors worldwide, with increasing incidence and mortality rates each year (1). This highly heterogeneous disease is driven by complex genetic and epigenetic alterations (2). In the era of precision oncology, molecular profiling has emerged as a powerful approach to guide targeted therapies through comprehensive genetic analysis (3, 4). Among key biomarkers, the BRAF gene serves as a critical predictor of survival. BRAF mutations—most notably the V600E variant—occur in approximately 10% of RC cases. This molecular subtype is characterized by aggressive early dissemination to the peritoneum and lymph nodes, as well as resistance to chemotherapy and patients often require more personalized treatment plans (5). Current clinical guidelines recommend anti-epidermal growth factor receptor (EGFR) therapy for BRAF wild-type colorectal cancer, whereas combination regimens incorporating BRAF inhibitors have demonstrated superior efficacy in BRAF-mutated cases (6–9). Although BRAF mutation occurs at a lower frequency compared to KRAS, it defines a subgroup of patients with extremely poor prognosis, aggressive tumor biology, and distinct resistance patterns to standard therapies.

Given the critical influence of BRAF status on treatment decisions, both the American Society of Clinical Oncology and the National Comprehensive Cancer Network recommend BRAF mutation testing at the time of initial diagnosis (7, 10). This approach not only facilitates personalized treatment planning but also aids in evaluating the risk of Lynch syndrome. Moreover, BRAF mutation has been established as an independent adverse prognostic factor and significantly reduced overall and progression-free survival in RC patients. Early identification of BRAF status is therefore essential for accurate risk stratification and treatment customization. However, conventional BRAF testing is generally conducted using postoperative resection specimens. In cases of advanced rectal cancer where surgery is not feasible, acquiring tissue samples for BRAF genetic analysis becomes particularly challenging. As a result, there is a pressing need for a noninvasive, preoperative method to predict BRAF status for early prognostic stratification, enabling clinicians to identify high-risk patients who may require intensified or alternative therapeutic strategies.

Magnetic resonance imaging (MRI) is indispensable in RC management, offering non-invasive diagnosis, staging, and prognosis prediction (11, 12). However, conventional MRI interpretation remains subjective and limited in molecular profiling. Amide proton transfer-weighted (APTw) MRI addresses this gap as a novel molecular imaging technique that generates contrast by detecting endogenous mobile proteins and peptides (13). Studies have applied APTw MRI to predict key prognostic markers in RC—including KRAS status, T stage, extramural vascular invasion, and treatment response,-highlighting its ability to characterize the tumor microenvironment and assess tumor heterogeneity non-invasively (14–19). Advanced image analysis methods, such as sub-regions or habitats, which can reveal intratumoral heterogeneity and K-means clustering for unsupervised voxel classification may further could improve outcome prediction across various cancers (20, 21).

Advanced image analysis methods, such as sub-region or habitat analysis that can reveal intratumoral heterogeneity, as well as K-means clustering for unsupervised voxel classification, may further improve outcome prediction across various cancers. Nevertheless, habitat-based APTw models for evaluating BRAF mutation status have not yet been reported.

Therefore, this study aims to investigate the value of habitat−derived APTw histogram features for the non-invasive prediction of BRAF mutation status in RC, and to evaluate its utility for prognostic stratification, in comparison and combine with diffusion-weighted imaging (DWI).

## Materials and methods

### Patient data

This prospective study was approved by the Ethics Review Committee of our hospital. The informed consent was obtained from all the enrolled patients (approval number: 2021K-133).

**Figure 1:**
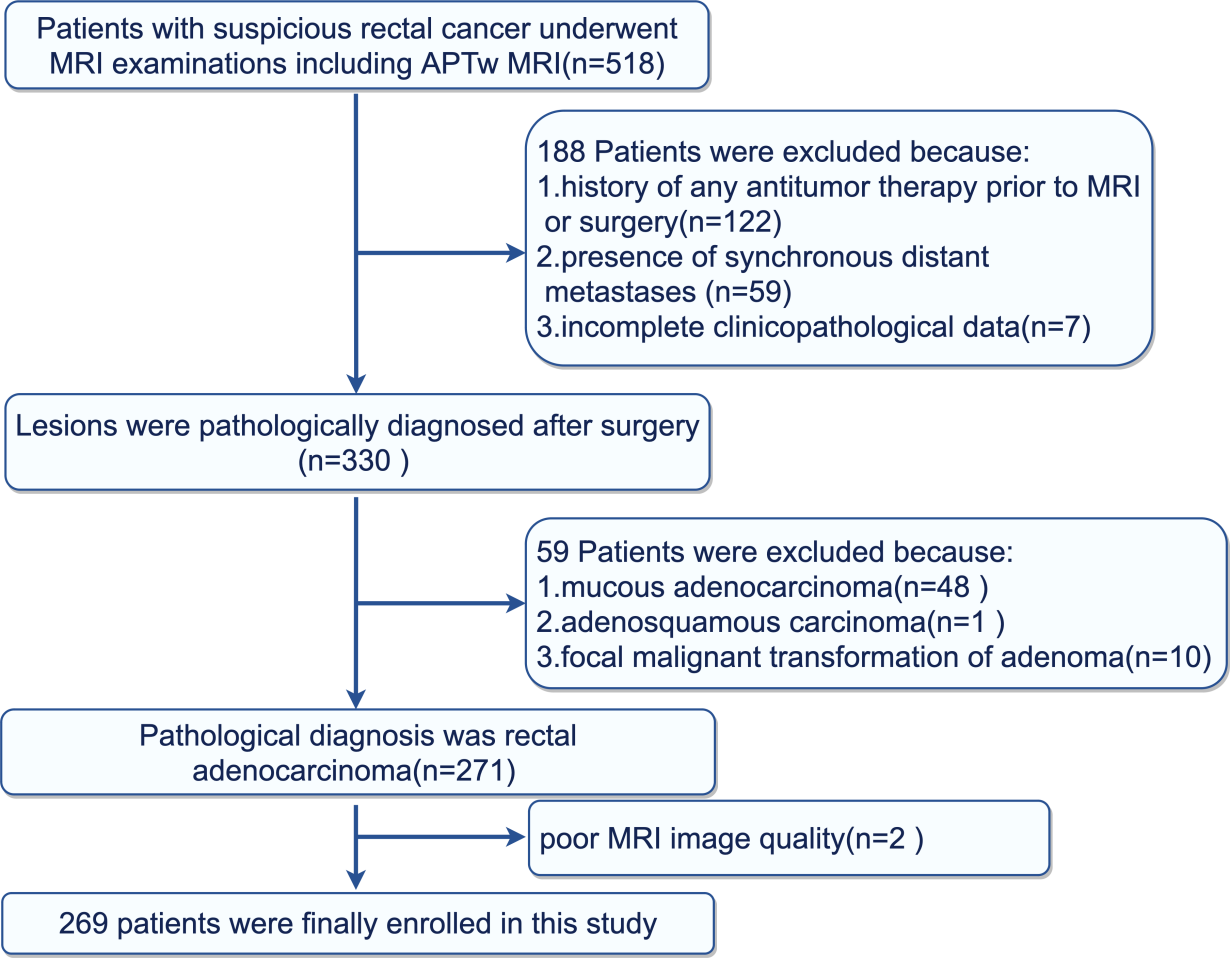
The process of patient recruitment.

From June 2021 to August 2025, a total of 518 consecutive RC patients who underwent surgical resection were initially enrolled. The inclusion criteria were as follows: pathologically confirmed rectal adenocarcinoma; preoperative pelvic MRI examination including conventional sequences, APTw imaging, and DWI; surgery performed within two weeks after the MRI scan; and availability of complete postoperative pathological records, including BRAF mutation status. The exclusion criteria were: history of any antitumor therapy (e.g., surgery, radiotherapy, or chemotherapy) prior to MRI or surgery; pathological diagnoses other than adenocarcinoma, such as mucinous adenocarcinoma or adenosquamous carcinoma; presence of synchronous distant metastases at diagnosis; and incomplete clinicopathological data. **Figure 1** shows the process of patient recruitment.

### MRI scanning protocol

All MRI examinations were conducted on a 3.0T system (Ingenia 3.0T, Philips Healthcare) using a 16-channel phased-array torso coil. The imaging protocol comprised the following sequences: Axial T1-weighted imaging (T1WI) and high-resolution T2-weighted imaging (HR-T2WI). DWI with b-values of 0 and 1000 s/mm², and 3D APTw imaging. Detailed acquisition parameters are summarized in **Table 1**.

**Table 1:** Main imaging parameters of the MRI protocol.

Parameters	3D-APTw imaging	DWI	T2WI
Sequence	TSE	EPI	TSE
Repetition time/echo time (msec)	6306/8.3	2893/65	4562/100
Flip angle (degree)	90	90	N/A
Field of view (mm2)	230×180×60	260×260×140	260×260×140
Spatial Resolution (mm3)	1.8×1.8×6	3×3×4	0.7×0.7×4
Matrix	128×100×6	88×86×4	372×324×4
section thickness (mm)	6	4	4
TSE factor	174	N/A	25
b Values (sec/mm2)	N/A	0,1000	N/A
Fat suppression	SPIR	SPIR	N/A
Acquisition time (min: sec)	5:23	1:59	3:12

N/A indicates not applicable. T2WI, T2-weighted imaging; APTw, amide proton transfer-weighted; DWI, diffusion-weighted imaging. TSE, turbo spin echo; EPI, echo planar imaging; SPIR, spectral presaturation with inversion recovery.

### Clinicoradiological data and follow-up

Clinical parameters (gender, age) and radiological characteristics, including TN stage, distance from the distal tumor edge to the anal verge, and extramural venous invasion (EMVI), were systematically collected from the institutional Picture Archiving and Communication System and electronic medical records.

Two abdominal radiologists (LLC. and L.Z., with 10 and 8 years of experience in rectal MRI, respectively), blinded to the molecular profiles, independently assessed all imaging features. Any interobserver discrepancies were resolved by a third senior radiologist (XY. L., with 20 years of experience).

All patients underwent regular postoperative follow-up via abdominal ultrasound, computed tomography (CT), or MRI. Tumor recurrence or metastasis was defined by the presence of typical imaging features on CT, MRI, or ultrasound, or by pathological confirmation. DFS was calculated from the date of surgery to the date of the first documented recurrence, metastasis, or the last follow-up. The follow-up period concluded on Jan. 1, 2026.

### Data analysis

Two radiologists (as mentioned above) identified the rectal lesions on T2WI and DWI images. They then manually delineated the entire tumor margin on the corresponding ADC and S0 maps to generate a volume of interest (VOI) using ITK-SNAP software, carefully avoiding necrotic or cystic areas and the intestinal lumen. The resulting VOIs were subsequently copied and co-registered to the APTw maps for feature extraction.

Based on these VOIs, histogram features from each ADC and APTw image were automatically extracted using Matlab software. The extracted features included first-order statistics such as minimum, maximum, mean, percentile, interquartile range, median, range, mean absolute deviation (MAD), robust mean absolute deviation (RMAD), root mean squared (RMS), energy, total energy, entropy, kurtosis, skewness, uniformity, and variance.

The inter-observer agreement was assessed by having both radiologists independently analyze the first 20 patients’ data. Intra-observer agreement was evaluated by the second radiologist (L.Z.) re-analyzing the same 20 cases after a 3-month interval to minimize recall bias. The feature set generated by the first radiologist was used for all subsequent statistical analyses.

### K-means clustering

**Figure 2:**
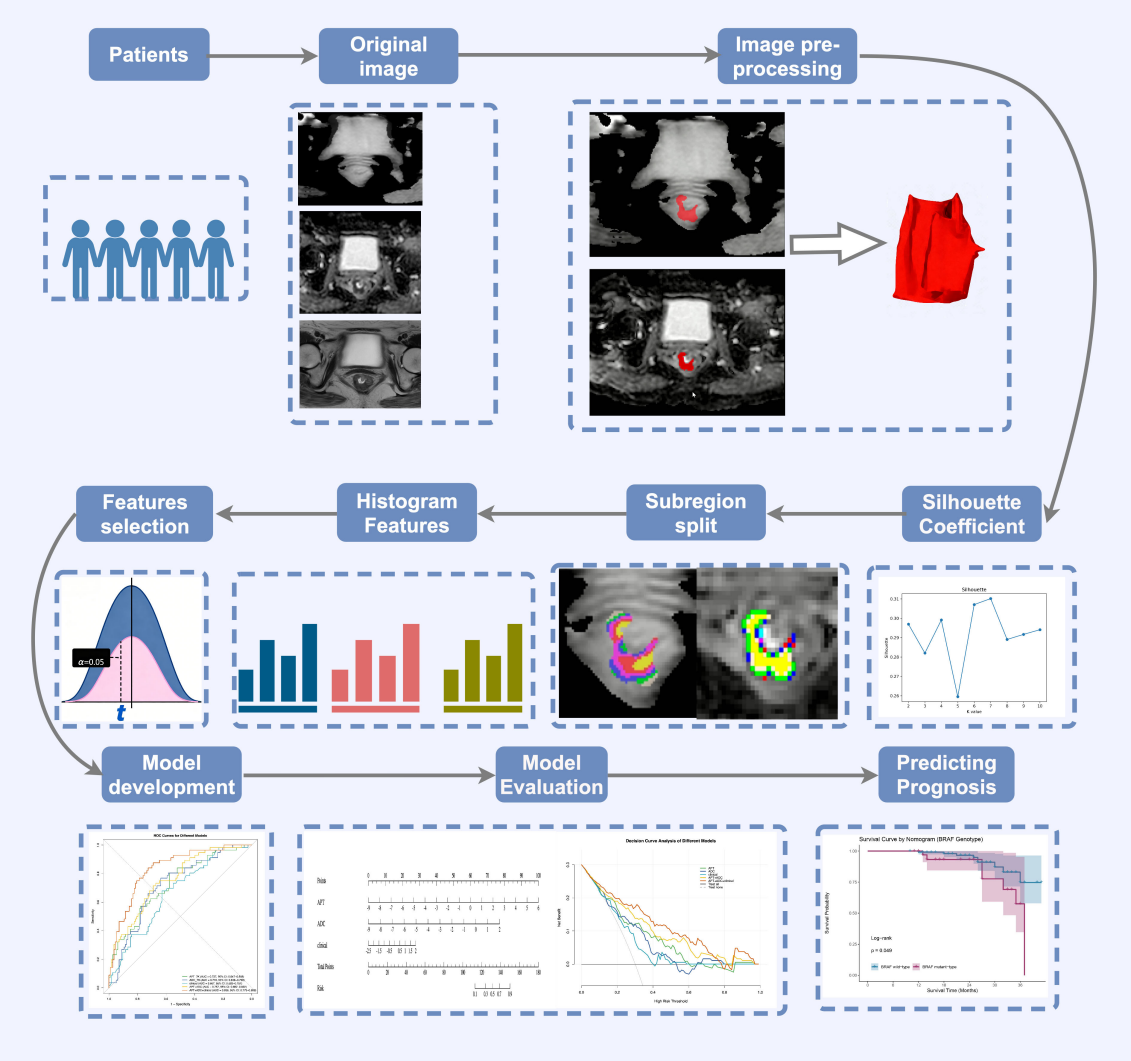
Flowchart of image analysis(by Figdraw).

K=means clustering was employed to segment the tumor into distinct subregions according to pixel values and distributions. Subsequently, sub-region histogram features were extracted from each of these subregions in the APTw and ADC maps (21, 22). The optimal number of clusters, K, was determined using the silhouette coefficient (23). Based on the evaluation of the silhouette coefficient for K values ranging from 2 to 10, we initially identified K = 5, 6, and 7 as the most promising candidate parameters and these three K values were further evaluated. The identified sub-regions were first sorted by ascending signal intensity. A t-test was then used to analyze differences in features between sub-regions. Stepwise regression was employed to eliminate multicollinearity and to screen for the final feature set. The selected features were used to construct a logistic regression classifier for discriminating the BRAF mutated from the wild-type group. The entire procedure is summarized in **Figure 2**. The detailed quantitative information regarding feature extraction, preliminary screening, and final feature selection across all habitat partition numbers (K, ranging from 3 to 7) are provided in Supplementary materials (**Supplementary Tables 1**, **S2**).

### Leakage-free train-test evaluation framework

The dataset was stratified by the outcome and randomly split into a training set (70%) and an independent test set (30%) using a fixed random seed (2024). To avoid information leakage, all data preprocessing, feature selection, and model training were performed exclusively within the training set, with the testing set used only once for final evaluation.

### Histopathology evaluation

An experienced gastrointestinal pathologist (J.L.), who was blinded to the MRI results, performed all pathological assessments. Following surgical resection, tumor tissues were processed according to standard histopathological procedures, including fixation, dehydration, and paraffin embedding. DNA was extracted from the resulting formalin-fixed, paraffin-embedded sections, and BRAF V600E mutation status was determined using a fluorescent polymerase chain reaction assay based on the amplification refractory mutation system.

### Statistical analysis

The normality of continuous variables was assessed using the Kolmogorov–Smirnov test. Normally distributed data are expressed as mean ± standard deviation, while non-normally distributed data are presented as median with interquartile range. Categorical variables are summarized as frequencies and percentages, and group comparisons were performed using the chi-square test or Fisher’s exact test, as appropriate. Inter- and intra-observer agreement for histogram parameters were evaluated using the intraclass correlation coefficient (ICC), interpreted as follows: <0.20, poor; 0.21–0.40, fair; 0.41–0.60, moderate; 0.61–0.80, good; and 0.81–1.00, excellent.

Differences in APT and ADC sub-regional histogram parameters between BRAF mutant and wild-type groups were analyzed using the independent samples t-test or Mann–Whitney U test, based on distribution normality and homogeneity of variance. The diagnostic performance of each model-clinical, habitat-based APTw/ADC, habitat-based APTw+ADC, and habitat-based APTw+habitat-based ADC+clinical—for predicting BRAF mutation status was evaluated using receiver operating characteristic (ROC) curve analysis. The sensitivity, specificity, and accuracy were calculated, and the areas under the curve (AUCs) were compared using DeLong’s test.

A nomogram was constructed to visualize the combined model (habitat-based APTw+ habitat-based ADC+clinical), and its calibration was assessed using the Hosmer–Lemeshow test. Decision curve analysis (DCA) was applied to evaluate the clinical utility of all the models. For survival analysis, the 2-year disease-free survival (DFS) rates were estimated using the Kaplan–Meier method and compared with the log-rank test. Variables with *P* < 0.05 in univariate Cox regression were entered into a multivariate Cox regression model to identify independent predictors of DFS. All statistical analyses were performed using SPSS (version 25.0) and R (version 4.2.3) software. A two-sided *P*-value < 0.05 was considered statistically significant.

## Results

### Baseline demographic and clinical characteristics of patients

According to the above selection criteria, a total of 269 patients with RC were included in this prospective study, 151 men as well as 118 females, with an average age of 62.30 ± 11.59 years old. According to their BRAF status, patients were categorized into a wild-type group (n=214) and a mutant group (n=55). Demographic and clinical characteristics of the two groups are summarized in **Table 2**. No significant differences were observed between the wild-type and mutant groups in terms of sex, age, tumor location, or differentiation grade (all *P*>0.05). However, significant differences were found in T/N stage between the two groups (*P* < 0.05). The independent test set contained 81 patients, including 16 BRAF-mutant cases and 65 BRAF wild-type cases.

**Table 2:** The demographic and clinical characteristics of the two groups.

Characteristic	Wild type(n=214)	Mutant type(n=55)	*P*
Age, years (Mean ± SD)	61.45 ± 12.34	62.01 ± 10.50	0.671
Gender,n (%)			0.289
Male	129(60.5%)	34(61.8%)	
Female	85(39.5%)	21(38.2%)	
Histologic grade,n (%)			0.301
Well	36(16.7%)	8(14.5%)	
Moderate	173(80.7%)	41(74.5%)	
Poor	5(2.6%)	6(10.9%)	
Tumor location,n (%)			0.183
Lower	58(27.2%)	20(36.4%)	
Middle	119(55.3%)	27(49.1%)	
Upper	37(17.5%)	8(14.5%)	
MR T stage,n (%)			**0.025**
T1/2	139(64.9%)	22(40%)	
T3/4	75(35.1%)	33(60%)	
MR N stage,n (%)			**0.045**
N0	122(57.0%)	21(38.2%)	
N+	92(43.0%)	34(61.8%)	
MR EMVI,n (%)			
Negative	160(74.6%)	32(58.2%)	0.065
Positive	54(25.4%)	23(41.8%)	

Chi-squared or Fisher’s exact tests, as appropriate, were used to compare the differences in categorical variables, while independent samples t test was used to compare the differences in age. Bold value: Rectal cancer with more advanced TN stage are prone to involve mutant BRAF (P = 0.025, 0.045, respectively).

EMVI, Extramural venous invasion.

### Univariate analysis of clinical predictors

Among the seven clinical variables tested, only MR T stage (OR = 3.16, 95% CI: 1.50–6.66, P = 0.02) and MR N stage (OR = 2.44, 95% CI: 1.25–4.76, P = 0.042) showed statistically significant associations with BRAF mutation. Other variables were not significant (all P > 0.05). The full univariate analysis results are provided in **Supplementary Table 3** (supplement materials).

### Comparison of sub-regional histogram features of APT and ADC maps for tumors with BRAF mutation and wild-type status

The ICC values of the inter- and intraobserver reproducibility ranged from 0.859 to 0.932 for all the measurements. Following differential analysis and stepwise regression, significant subregional histogram features were identified from the APTw maps(**Table 3**). At K = 7, uniformity in subregions 1, 2, and 6 was significantly lower in the BRAF-mutated group than in the wild-type group (*P* < 0.05). Additionally, several APTw histogram features in subregions 1, 5, and 6 also showed significant differences between the two groups (*P* < 0.05; **Table 3**).

**Table 3:** The significant features of APTw image sub-regions when K = 7.

Features of clusters(Sub-region)(Mean ± SD)	Mutant type(n=55)	Wild type(n=214)	*P*
Kurtosis(Sub-region 5)(Mean ± SD)	4.489 ± 3.361	3.488 ± 1.714	0.011
Skewness(Sub-region 1)(Mean ± SD)	0.032 ± 0.320	0.195 ± 0.429	0.014
Skewness(Sub-region 5)(Mean ± SD)	0.189 ± 0.839	-0.057 ± 0.602	0.030
Entropy(Sub-region 6)(Mean ± SD)	2.389 ± 0.475	2.196 ± 0.567	0.031
Uniformity(Sub-region 2)(Mean ± SD)	0.135 ± 0.047	0.155 ± 0.063	0.035
Uniformity(Sub-region 6)(Mean ± SD)	0.239 ± 0.087	0.277 ± 0.123	0.039
Uniformity(Sub-region 1)(Mean ± SD)	0.121 ± 0.043	0.138 ± 0.055	0.047

For ADC maps, at K = 7, the ADC percentile values and mean, min, RMS, skewness values in subregion 1 was significantly lower in the mutated group (*P* < 0.05). Kurtosis feature in subregion 2 also differed significantly between groups (*P* < 0.05; **Table 4**).

**Table 4:** The significant features of ADC image sub-regions when K = 7.

Features of clusters(Sub-region)(Mean ± SD)	Mutant type(n=55)	Wild type(n=214)	*P*
Kurtosis(Sub-region 2)	3.756 ± 2.701	3.131 ± 0.799	0.025
Mean(Sub-region 1)(Mean ± SD)	0.987 ± 0.274	1.091 ± 0.284	0.027
Minimum(Sub-region 1)(Mean ± SD)	0.716 ± 0.330	0.850 ± 0.311	0.011
Root.Mean.Square(Sub-region 1)(Mean ± SD)	0.993 ± 0.273	1.095 ± 0.281	0.026
Skewness(Sub-region 1)(Mean ± SD)	-0.296 ± 0.557	-0.125 ± 0.424	0.028
10th.Percentile(Sub-region 1)(Mean ± SD)	0.882 ± 0.284	0.989 ± 0.288	0.024
15th.Percentile(Sub-region 1)(Mean ± SD)	0.903 ± 0.279	1.008 ± 0.285	0.025
1th.Percentile(Sub-region 1)(Mean ± SD)	0.785 ± 0.309	0.905 ± 0.295	0.016
25th.Percentile(Sub-region 1)(Mean ± SD)	0.932 ± 0.274	1.035 ± 0.285	0.028
50th.Percentile(Sub-region 1)(Mean ± SD)	0.989 ± 0.270	1.089 ± 0.282	0.029
5th.Percentile(Sub-region 1)(Mean ± SD)	0.850 ± 0.291	0.961 ± 0.291	0.022
75th.Percentile(Sub-region 1)(Mean ± SD)	1.046 ± 0.279	1.147 ± 0.285	0.030
85th.Percentile(Sub-region 1)(Mean ± SD)	1.074 ± 0.285	1.176 ± 0.292	0.033
90th.Percentile(Sub-region 1)(Mean ± SD)	1.092 ± 0.291	1.195 ± 0.297	0.035
95th.Percentile(Sub-region 1)(Mean ± SD)	1.117 ± 0.301	1.221 ± 0.302	0.036
99th.Percentile(Sub-region 1)(Mean ± SD)	1.162 ± 0.325	1.269 ± 0.318	0.044
Maximum(Sub-region 1)(Mean ± SD)	1.205 ± 0.348	1.314 ± 0.328	0.048

### ROC curve analysis

**Figure 3:**
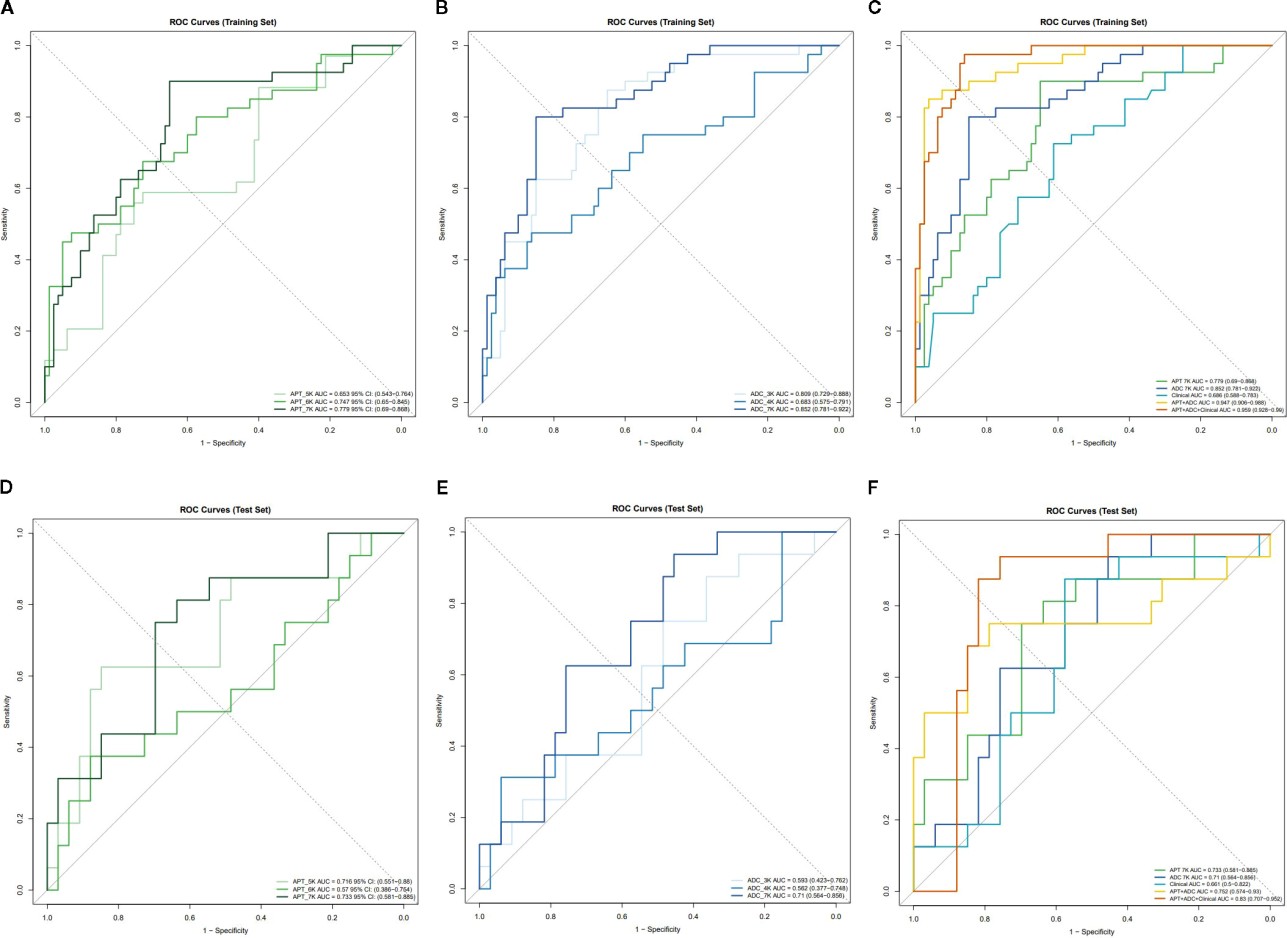
The ROC curves of the APT model **(A)**,ADC model **(B)**, APT+ADC model, clinical mode, and combined model **(C)** for predicting BRAF mutation for the training set; **(D–F)** for testing set.

Logistic regression was employed to differentiate between BRAF mutated and wild-type cases using the identified habitat-based APTw model. The diagnostic performance at different cluster numbers (K) was as follows: at K = 5, the AUC was 0.716 (95%CI: 0.551-0.88), with a sensitivity of 77.8% and specificity of 44.8%; at K = 6, the AUC was 0.57 (95%CI: 0.386-0.754), sensitivity 64.2%, and specificity 50.8%; and at K = 7, the model achieved an AUC of 0.733 (95%CI: 0.581-0.885), with 81.2% sensitivity and 63.6% specificity. For ADC, K = 7 was optimal for differentiation, the AUC, sensitivity, and specificity were 0.71 (95%CI: 0.564-0.856), 93.8%, and 45.5%in testing set. (**Figure 3**).

Univariate logistic regression identified MR T stage and MR N stage as potential clinical predictors of BRAF mutation (all *P* < 0.05). A clinical model constructed with these predictors yielded an AUC of 0.661 (95%CI: 0.5-0.822).

A combined model integrating APTw/ADC habitat based-histogram features with MR T/N stage achieved an AUC of 0.83 (95%CI: 0.707-0.952). The predictive performance of all models in the training and testing sets—APTw, ADC, clinical, APTw+ADC, and the combined model—is summarized in **Table 5** and **Figure 3**. DeLong’s test indicated that the combined model significantly outperformed the other models (*P* < 0.05). The detailed results have been provided as a supplementary **Supplementary Table S4**.

**Table 5:** The Performance of the each models in predicting BRAF mutation.

Model	AUC (95%CI)	Accuracy (95%CI)	Sensitivity (95%CI)	Specificity (95%CI)	PPV (95%CI)	NPV (95%CI)	Cut off
Training set							
APTw(k=7)	0.779(0.69-0.868)	0.767(0.648-0.865)	0.825(0.782 - 0.890)	0.738(0.517 - 0.818)	0.611 (0.537 - 0.686)	0.894 (0.749 - 0.934)	0.292
ADC(k=7)	0.852(0.781-0.922)	0.833(0.704-0.870)	0.8(0.649 - 0.856)	0.85 (0.757 - 0.898)	0.727 (0.671 - 0.824)	0.895 (0.805 - 0.939)	0.15
clinical model	0.686 (0.568-0.777)	0.65(0.578-0.768)	0.725(0.639 - 0.817)	0.613(0.556 - 0.701)	0.483(0.314 - 0.681)	0.817(0.666 - 0.884)	0.323
APTw+ADC	0.947(0.906-0.988)	0.925(0.767-0.984)	0.85(0.763 - 0.904)	0.963 (0.763 - 0.994)	0.919(0.798 - 0.939)	0.928 (0.770 - 0.951)	0.518
APTw+ADC+clinical	0.959(0.928-0.99)	0.9 (0.779-0.955)	0.975(0.805 - 0.996)	0.863(0.778 - 0.902)	0.78 (0.659 - 0.876)	0.986(0.778 - 0.999)	0.229
Testing set							
APTw(k=7)	0.733(0.581-0.885)	0.694(0.548-0.788)	0.812(0.682 - 0.840)	0.636(0.517 - 0.768)	0.52(0.337 - 0.686)	0.875 (0.749 - 0.904)	0.238
ADC(k=7)	0.71(0.564-0.856)	0.612(0.504-0.740)	0.938(0.749 - 0.966)	0.455 (0.357 - 0.678)	0.455(0.371 - 0.624)	0.938(0.805 - 0.989)	0.126
clinical model	0.661 (0.574-0.93)	0.673(0.568-0.817)	0.775(0.639 - 0.917)	0.576(0.456 - 0.751)	0.5(0.414 - 0.681)	0.805(0.766 - 0.884)	0.325
APTw+ADC	0.752(0.574-0.93)	0.776(0.667-0.844)	0.75(0.663 - 0.874)	0.788(0.686 - 0.878)	0.632(0.508 - 0.819)	0.867(0.670 - 0.931)	0.524
APTw+ADC+clinical	0.83(0.707-0.952)	0.816(0.713-0.891)	0.938(0.805 - 0.976)	0.785(0.666 - 0.877)	0.517(0.325 - 0.706)	0.981(0.897 - 0.999)	0.518

APTw, amide proton transfer-weighted; ADC, apparent diffusion coefficient; AUC, area under the ROC curve; 95% CI, 95% confidence interval; NPV, negative predictive value; PPV, positive predictive value.

The test set comprised 81 patients, including 16 BRAF-mutant (19.8%) and 65 BRAF wild-type (80.2%) cases, mirroring the prevalence in our training cohort. The model correctly identified 15 out of 16 mutant mutant patients, while 1 mutant patients were missed. The confusion matrix yielded 15 true positives, 1 false negative, 51 true negatives, and 14 false positives. Therefore, the recall (sensitivity) for the minority class is 93.8%.

### Clinical use (nomogram construction and apparent performance)

**Figure 4:**
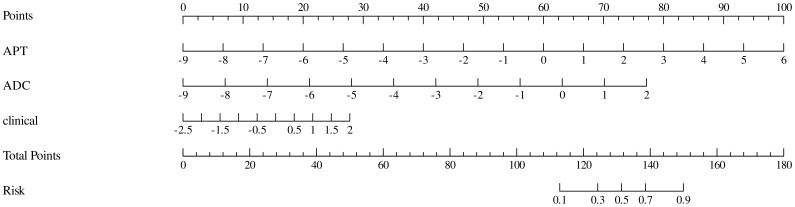
The developed APTw+ ADC +clinical factors nomogram to predict BRAF mutation.

The combined model was presented as a nomogram for clinical use (**Figure 4**).

**Figure 5:**
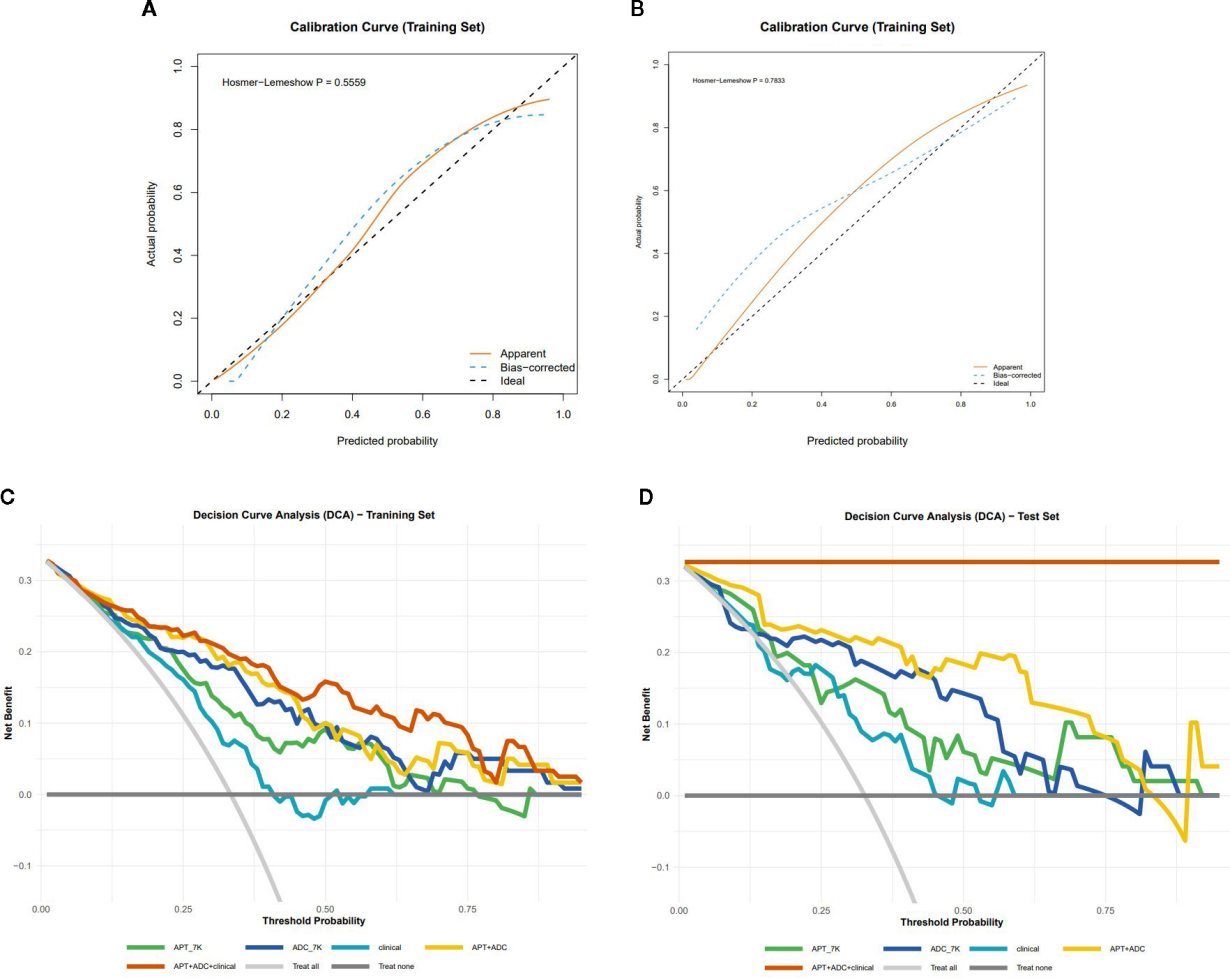
The calibration curve for predicting BRAF mutation status **(A, B)** for the training and testing set; the DCA for predict BRAF mutation using different models **(C,D)** for the training and testing set.

The calibration curves of the integrated nomogram to predict BRAF mutation of nomogram demonstrated good agreement. Good calibration was observed, and the Hosmer-Lemeshow test showed that the final model achieved acceptable and reliable calibration with well-controlled subgroup-level predictive deviation(*P*>0.05) (**Figures 5A, B** and supplementary materials **Supplementary Table 5**). DCA demonstrated that the nomogram provided superior clinical net benefit compared to the “treat all” or “treat none” strategies when the threshold probability ranged from approximately 0.1 to 0.75. These results indicate that the nomogram, incorporating APT, ADC, MR-TN stage serves as a reliable and clinically useful tool for predicting BRAF mutation in patients with RC(**Figures 5C, D**).

### Prognostic analysis of the optimal model

**Figure 6:**
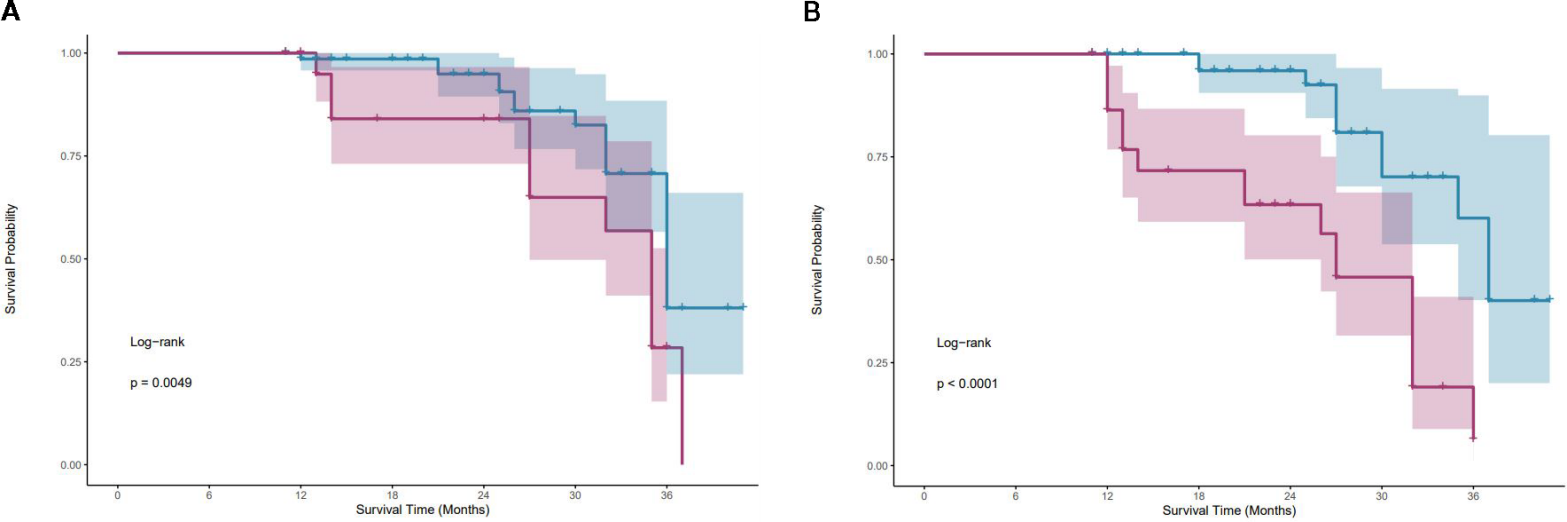
Kaplan–Meier survival curves according to pathological BRAF mutation **(A)** and nomogram-predicted BRAF mutation in the whole cohort **(B)** for testing set.

Follow-up data were available for 256 patients, with 13 cases lost to follow-up. The median follow-up period for the entire cohort was 25 months (range: 10–48). The BRAF mutation group exhibited a significantly worse 2-year DFS rate compared to the wild-type group (81.13% vs. 94.17%, *P* = 0.049). Kaplan–Meier analysis further demonstrated that patients stratified into the mutation group by the nomogram had significantly worse DFS than those in the wild-type group (*P* < 0.05) (**Figure 6**). Univariate and multivariate Cox proportional hazards analyses identified the nomogram score as a significant risk factor for 2-year DFS (hazard ratio [HR]=2.65, 95% CI: 1.09-7.25,*P* < 0.05).

## Discussion

With advances in precision medicine, BRAF mutation status has become relevant for clinical decision-making in RC (10). While histopathology remains the gold standard for tumor diagnosis and classification, the limitations of tissue-based genetic testing—such as intra-tumor heterogeneity, clonal evolution, and poor DNA quality, particularly in biopsy samples—are increasingly recognized (3). These issues may result in an incomplete genetic profile of the tumor. Thus, we aimed to identify an effective imaging-based approach by using habitat-based quantitative APT/ADC features to differentiate between mutant and wild-type BRAF genotypes in RC.

The analysis revealed significant differences in subregional histogram features derived from APTw and ADC maps between BRAF mutant and wild-type groups, at the optimal cluster number (K = 7), with similar AUCs of 0.733 and 0.71, respectively. By integrating these imaging features with clinical factors, we constructed a combined nomogram that demonstrated superior predictive performance for preoperative BRAF mutation status (AUC = 0.83) than other models. With a false negative rate of 6.2%, our framework successfully detects the vast majority of mutation-positive patients in an unseen cohort, supporting its potential utility as a reliable triage tool. Furthermore, the nomogram was significantly associated with 2-years DFS (HR = 2.63), supporting its potential role in preoperative risk stratification and prognostic assessment.

In our investigation, APT subregional histogram features (Kurtosis, Skewness, Entropy) were significantly higher in the BRAF mutant group compared with wild-type tumors, while uniformity values were lower. APT imaging reflects the concentration of mobile proteins and peptides in tissue, with signal intensity primarily determined by cellular density, cytosolic protein content, and intracellular pH (24–26). These higher histogram features quantitatively capture the greater structural disorder and biological aggression inherent to BRAF-mutant tumors, which manifest as a more heterogeneous and extreme distribution of mobile proteins and peptides detectable by APTw MRI. In addition, lower uniformity quantitatively confirms a less homogeneous and more disordered spatial distribution of mobile proteins and peptides, which is a hallmark of the biological aggressiveness and structural chaos driven by the BRAF mutation (27).

In this study, we identified a significant correlation between several histogram features derived from ADC values and BRAF mutations. Theoretically, tumors harboring such mutations tend to exhibit greater aggressiveness and enhanced angiogenesis, which contribute to more rapid progression and poorer survival outcomes (28). These pathological characteristics often correspond to restricted water diffusion, reflected as lower ADC values (29).

This underlying mechanism may explain the relatively high genotype prediction performance achieved by DWI, as it captures functional tissue information by quantifying water molecular mobility through the ADC metric.

Previous studies have suggested that CT radiomic features, PET habitat-derived parameters, or MRI radiomic characteristics may hold potential for predicting BRAF mutation status in patients with RC (30–32). However, these studies typically defined positivity based on mutations in any of the KRAS, NRAS, or BRAF genes—where one or more mutations classified a case as positive (31, 32). This approach may complicate the clinical interpretation and application of their findings. Furthermore, previous research has focused broadly on colorectal cancer without distinguishing between colon and RC (30, 32). Combining these two distinct cancer types could obscure critical differences in their optimal clinical management and limit the applicability of the findings specifically to RC patients.

This study is the first to explore the predictive value of APTw MRI for BRAF mutation status in RC. Our model, integrating subregional APT/ADC histogram features with clinical factors, achieved favorable performance in identifying BRAF mutations, underscoring the utility of habitat analysis techniques in characterizing complex tumor microenvironments. This approach is particularly effective in capturing intricate intratumoral heterogeneity, as it clusters voxels with similar characteristics to delineate metabolic variations driven by genetic diversity, thereby enabling a finer-grained assessment of the tumor microenvironment (33).

The advantage of this habitat-driven approach in quantifying tumor heterogeneity has also been supported by several previous studies (18, 34). For instance, Xie et al (18). utilized APTw subregional histogram features to identify heterogeneous tumor regions for predicting tumor budding grade, while Cai et al. (34) developed a multiparametric MRI-based subregional model to assess high-risk areas related to microsatellite instability status.

These results demonstrate the capability of habitat imaging to capture localized pathological and molecular characteristics. Moreover, compared to CT or PET/CT, MRI offers distinct advantages in this context, including the absence of ionizing radiation, no need for contrast agent injection in APTw imaging, and superior soft tissue contrast—all of which support its role as a preferable modality for noninvasive molecular characterization.

In our study, univariate logistic regression identified MR T/N stage as independent clinical predictors of BRAF mutation. In clinical practice, integrating multiple biomarkers often yields better predictive performance than relying on any single marker. Accordingly, we combined the APT and ADC features with the clinical factors, which further improved predictive efficacy. The resulting nomogram visually summarizes this combined model and demonstrated the highest discriminative ability among all evaluated approaches.

Multiple studies have established that BRAF mutation is associated with unfavorable prognosis in patients with RC (8, 35, 36). Consistent with these findings, our results confirmed that BRAF mutation status significantly correlates with worse 2-year DFS. Uni-and multivariate Cox regression analyses further identified the nomogram score as an independent prognostic factor for DFS. Given the significantly worse outcomes observed in the mutant group, our nomogram may assist in optimizing treatment strategies by enabling timely intervention with more intensive or alternative regimens, thereby potentially reducing unnecessary treatment-related toxicity and improving survival. These findings support the clinical utility of the nomogram in facilitating risk-adapted treatment planning and promoting personalized therapeutic approaches.

Several limitations should be considered in this study. First, this work represents a preliminary investigation with a relatively small sample size. Therefore, these findings should be viewed as a proof-of-concept that requires prospective multi-center validation with center-effect harmonization before any clinical translation. Second, our analysis focused solely on histogram-based features from APTw images. Other radiomic feature classes, such as texture and transform-based features, may capture more comprehensive tumor characteristics and should be incorporated in future investigations. Third, although manual segmentation remains common in histogram-based studies, it is subject to interobserver variability. The implementation of automatic segmentation methods could further improve the robustness and efficiency of radiomic analyses for predicting BRAF mutations in RC. Fourth, only the BRAF mutation status was considered in this study; the interaction between BRAF mutation and the KRAS mutation status need to be further considered. Fifth, our model is primarily applicable to patients undergoing direct surgery and that external validation in neoadjuvant-treated cohorts is needed. In the end, the model did not explicitly address class imbalance, and the modest number of mutant cases may affect its stability and reproducibility, which should be assessed in future studies.

## Conclusion

Habitat-based APTw/ADC histogram features, combined with clinical predictors model demonstrates good performance in preoperatively predicting BRAF mutation status and clinical outcomes in RC patients. This noninvasive preoperative tool enables improved risk stratification prior to treatment initiation, thereby providing valuable support for clinical decision-making and facilitating personalized therapeutic strategies.

## Data Availability

The original contributions presented in the study are included in the article/**Supplementary Material**. Further inquiries can be directed to the corresponding authors.
