# Supplementary material for: Habitat-based amide proton transfer-weighted MRI model for predicting BRAF mutation and prognostic stratification in rectal cancer
Source: Front Oncol. 2026 Jul 3;16:1862322. doi: 10.3389/fonc.2026.1862322 (PMC13375517; doi:10.3389/fonc.2026.1862322)
Supplement: Supplementary file 1 [file Table1.docx]

1. **Feature Selection and Multicollinearity Analysis**

First-order histogram radiomic features were extracted from APTw and ADC maps for each habitat partition number *K* (3–7). For each *K*, 25 features were extracted per subregion, and the total initial feature number was calculated as 25 × *K*.

Univariate Student’s *t*-test was used for preliminary feature screening, and features with *P* < 0.05 were retained for subsequent modeling. Bidirectional stepwise regression was then performed to select the optimal feature set. The regression criteria were set as follows: variables with *P* < 0.05 were allowed to enter the model, while variables with *P* > 0.10 were removed. The Akaike Information Criterion (AIC) was used to evaluate model fitting. At each iteration, both backward elimination of existing variables and forward re-inclusion of previously excluded variables were tested. The iteration stopped when no variable adjustment could reduce the AIC, and the model with the minimum AIC was determined as the final model.

Multicollinearity among predictors was assessed using the variance inflation factor (VIF). A cutoff of VIF > 10 was applied to identify severe multicollinearity, and corresponding features were excluded. For models with fewer than two independent variables, VIF analysis was not conducted, as multicollinearity was theoretically absent. The number of features at each screening stage and VIF results of all final models are summarized in Supplementary Table S1. Detailed iteration procedures of stepwise regression are shown in Table S2.

| Model | Initial extracted features | Features after t-test (P<0.05) | Features after stepwise regression | Final retained features | Final AIC | Multicollinearity (VIF) |
| --- | --- | --- | --- | --- | --- | --- |
| APT (K=5) | 125 | 4 | 2 | 2 | 202.9 | — |
| APT (K=6) | 150 | 11 | 2 | 2 | 209.38 | — |
| APT (K=7) | 175 | 7 | 4 | 4 | 203.9 | VIF range: 1.05–1.09 (< 10) |
| ADC (K=3) | 75 | 15 | 4 | 3 | 214.11 | One feature with VIF=425.32 (>10) excluded |
| ADC (K=4) | 100 | 6 | 2 | 2 | 215.66 | — |
| ADC (K=7) | 175 | 17 | 5 | 4 | 210.44 | One feature with VIF=32.18 (>10) excluded |
| Clinical model | 7 | 2 | 2 | 2 | 211.66 | — |
| APT7K + ADC7K | — | 24 | 7 | 4 | 209.38 | Features with VIF >10 excluded |
| APT7K+ADC7K+Clinical | — | 26 | 9 | 9 | 200.35 | Features with VIF >10 excluded |

**Table S1 The number of features at each screening stage for all models**

**Table S2 The detailed process of** **Stepwise Regression**

| Iteration | Model composition (features) | Number of features | AIC | Main operation |
| --- | --- | --- | --- | --- |
| Initial | Kurtosis.5, Skewness.1, Skewness.5, Entropy.6, Uniformity.2, Uniformity.6, Uniformity.1 | 7 | 209.57 | Baseline model |
| Step 1 | Kurtosis.5, Skewness.1, Skewness.5, Entropy.6, Uniformity.2, Uniformity.6 | 6 | 207.58 | Removed Uniformity.1 |
| Step 2 | Kurtosis.5, Skewness.1, Skewness.5, Uniformity.2, Uniformity.6 | 5 | 205.58 | Removed Entropy.6 |
| Step 3 (Final) | Kurtosis.5, Skewness.1, Skewness.5, Uniformity.6 | 4 | 203.9 | Removed Uniformity.2; Iteration terminated |

**Table S3. Univariate logistic regression analysis of clinical variables associated with BRAF mutation status**

| Variable | Category / Unit | Β (coefficient) | SE | OR | 95% CI | P value |
| --- | --- | --- | --- | --- | --- | --- |
| Age (years) | Continuous | 0.01 | 0.01 | 1.01 | (0.99–1.03) | 0.45 |
| Gender | Male vs. female | 0.23 | 0.32 | 1.26 | (0.67–2.36) | 0.47 |
| Histologic grade | Well vs.Moderate vs.Poor | 0.31 | 0.33 | 1.57 | (0.78–3.13) | 0.28 |
| Tumor location | Lower vs. middle/upper | 0.42 | 0.35 | 1.52 | (0.76–3.03) | 0.23 |
| MR T stage | T3–4 vs. T1–2 | 1.15 | 0.38 | 3.16 | (1.50–6.66) | **0.02** |
| MR N stage | N+ vs. N0 | 0.89 | 0.34 | 2.44 | (1.25–4.76) | **0.042** |
| MR EMVI | Negative vs. Positive | 0.57 | 0.33 | 1.77 | (0.93–3.36) | 0.08 |

**Table S4 DeLong test for pairwise comparison of AUCs in testing set**

| Comparison | AUC difference | Standard error | Z statistic | *P* value |
| --- | --- | --- | --- | --- |
| Combined model vs. APT-only model | 0.097 | 0.028 | 2.545 | 0.007 |
| Combined model vs. ADC-only model | 0.12 | 0.027 | 2.591 | 0.009 |
| Combined model vs. APTw+ADC | 0.078 | 0.021 | 2.143 | 0.042 |
| Combined model vs. Clinical model | 0.169 | 0.039 | 3.632 | 0.001 |

1. **Calibration Analysis**

To systematically evaluate and optimize model calibration, and verify the stability of calibration performance under different shrinkage intensities, we set gradient penalty values covering zero, low, and moderate shrinkage levels (penalty = 0, 0.05, and 0.2). The penalty values of 0.05 and 0.2 were selected based on the standard parameter setting range for penalized radiomic prediction models: 0.05 represented mild regularization to slightly suppress overfitting, while 0.2 represented moderate regularization to further correct high-dimensional feature-induced miscalibration, which covered the most commonly used and effective shrinkage intervals for clinical radiomic models. The full calibration indices under different shrinkage intensities are listed as follows:

Original unpenalized model (penalty = 0):

Calibration intercept = −2.6601, calibration slope = 5.3468, Brier score = 0.1539, ECE = 0.0885

Light shrinkage model (penalty = 0.05):

Calibration intercept = −2.6897, calibration slope = 5.4902, Brier score = 0.1614, ECE = 0.0685

Final optimized shrinkage model (penalty = 0.2):

Calibration intercept = −2.6778, calibration slope = 5.4769, Brier score = 0.1643, ECE = 0.0623

We observed that the calibration intercept and slope remained relatively stable across different penalty values and could not fully approach the ideal values of 0 and 1. This phenomenon is common in radiomic prediction models containing a large number of highly correlated histogram-derived features, which inevitably cause systemic probability stretching and limit the further optimization of intercept and slope. Nevertheless, with the increase of shrinkage penalty, the ECE decreased continuously from 0.0885 to 0.0623, indicating progressively improved subgroup-level calibration with a final average predictive error of only 6.23%. Compared with mild regularization (penalty = 0.05), moderate regularization (penalty = 0.2) achieved the lowest ECE value, realizing the optimal subgroup calibration performance without a substantial increase in prediction error. The Brier score slightly increased within a narrow and acceptable range (0.1539–0.1643), which represents a moderate and stable overall prediction error typical for radiomics-based models.

Collectively, after optimized penalized shrinkage correction (penalty = 0.2), combined with the non-significant Hosmer-Lemeshow test, the final model achieved acceptable and reliable calibration with well-controlled subgroup-level predictive deviation.

**Table S5 The results of Calibration**

| Calibration index | Original(penalty=0) | penalty=0.05 | penalty=0.2 | Ideal value |
| --- | --- | --- | --- | --- |
| Calibration intercept | −2.6601 | −2.6897 | −2.6778 | ≈0 |
| Calibration slope | 5.3468 | 5.4902 | 5.4769 | ≈1 |
| Brier score | 0.1539 | 0.1614 | 0.1643 | Close to 0 |
| ECE | 0.0885 | 0.0685 | 0.0623 | Close to 0 |
